# Lymphomatoid papulosis type E: An indolent diagnosis in disguise

**DOI:** 10.1111/1346-8138.17642

**Published:** 2025-01-20

**Authors:** William Tuckwell, Jenny Callander, Krishnakumar Subramanian, Patrick Yesudian, Paul D. Yesudian

**Affiliations:** ^1^ School of Medicine Cardiff University Cardiff UK; ^2^ Aneurin Bevan University Health Board Newport UK; ^3^ Vision Research Foundation Chennai India; ^4^ Private Practice Chennai India; ^5^ Wrexham Maelor Hospital Wrexham UK

**Keywords:** CD8, lymphocytes, lymphomatoid papulosis, methotrexate, ulcers

A 51‐year‐old Indian man presented with a 2‐month history of foot ulcers causing pain on exertion. Examination revealed two shallow, oval ulcers on the right dorsal foot with central eschars (Figure 1a). Inspection of the surrounding skin showed atrophic scarring, suggesting previously healed lesions. The patient had no systemic symptoms and no palpable lymphadenopathy. A diagnosis of vasculitis was considered.

**FIGURE 1:**
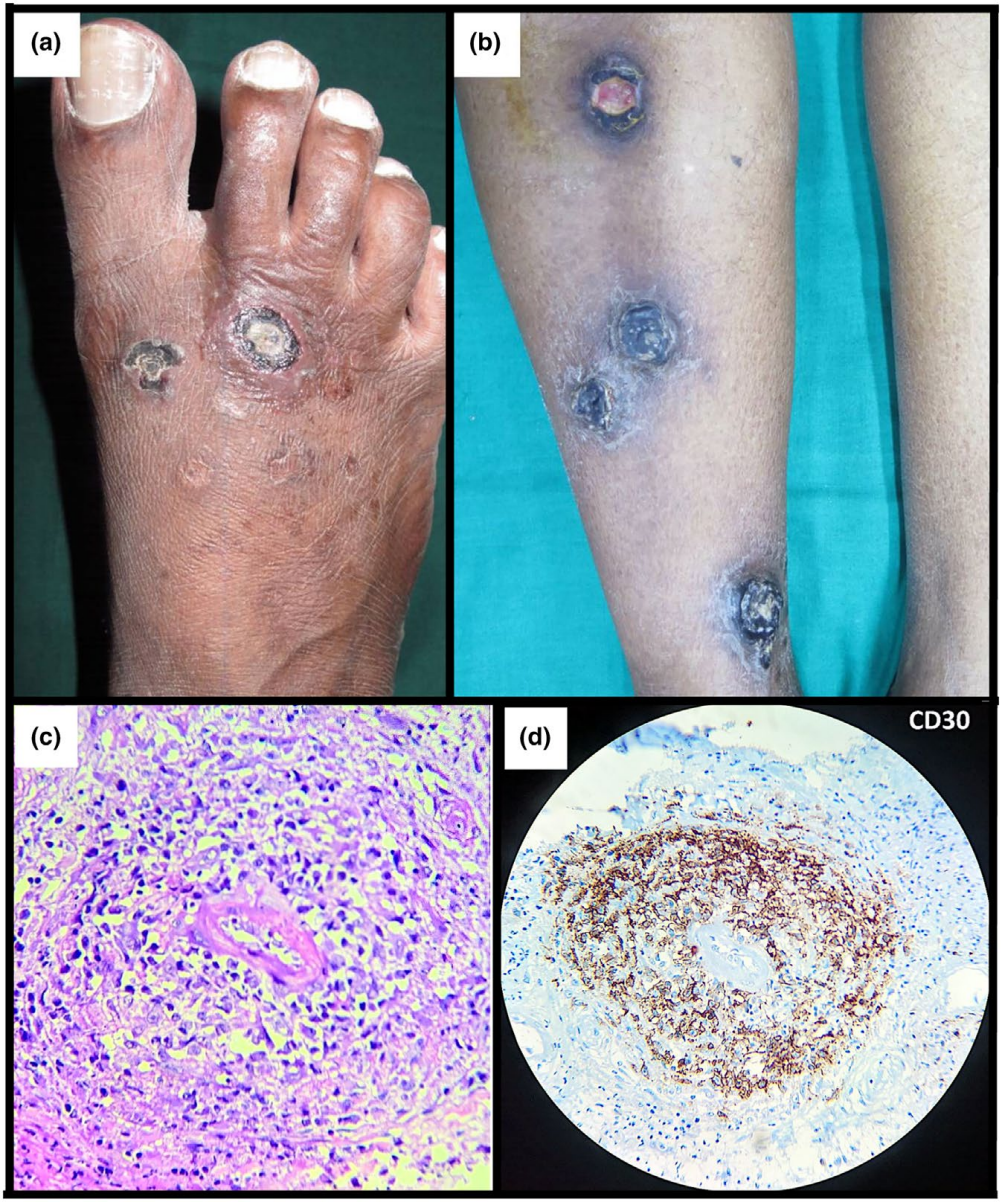
(a) Initial presentation of two shallow, oval ulcers on the dorsum of the right foot. (b) Four new lesions on the right anterior shin, presenting 3 months after the first encounter. (c) Atypical lymphocytes showing an angiocentric distribution around the vessels. (d) Atypical angiocentric lymphocytes staining positive for CD30 protein. Staining: (c) Hematoxylin and eosin, original magnification x20; (d) DAB chromogen, original magnification, ×20.

The patient presented 3 months later with four new ulcerated and crusted plaques on the right anterior shin (Figure 1b). The previously identified lesions on the right foot had resolved. A skin biopsy of one of the new lesions was performed.

Histology revealed widespread epidermal and dermal necrosis with central ulceration. In the dermis, histiocytes, eosinophils, and infiltrates of atypical lymphocytes were found in an angiocentric pattern (Figure 1c). Lymphocytes invading the vessel walls had led to angiodestruction. Immunohistochemical investigations showed that CD2, CD3, CD4, CD5 and CD7 were positive. Additionally, CD30 and CD8 were focally positive (Figure 1d).

These findings supported a diagnosis of lymphomatoid papulosis (LyP) type E and treatment was commenced with methotrexate. The ulcers resolved within 6 weeks. Treatment was continued for 12 months and no further lesions were observed.

Lymphomatoid papulosis is a rare lymphoproliferative disorder characterized by recurrent papulo‐nodular lesions.^1^ Despite low disease‐specific mortality rates, LyP carries a 20% risk of developing secondary lymphoid malignancy.^2^

Lymphomatoid type E is an uncommon subtype, making up less than 5% of cases.^3^ It is characterized clinically by lesions that rapidly break down to form large, eschar‐like, necrotic ulcers; the other five subtypes differ as smaller superficial ulceration is seen. Ulceration typically resolves spontaneously within 3–6 weeks leaving atrophic varioliform scarring. Histopathological distinction is based on angiocentric CD30+ and CD8+ atypical infiltrates.^4^ Contradictory to its aggressive presentation, LyP type E has an excellent prognosis; only 5% of patients develop a secondary malignant lymphoma.^1^

## CONFLICT OF INTEREST STATEMENT

None declared.

## CONSENT

Informed consent was provided by the patient for the use of clinical images.
